# Diffusion Tensor Imaging Evaluation of Neural Network Development in Patients Undergoing Therapeutic Repetitive Transcranial Magnetic Stimulation following Stroke

**DOI:** 10.1155/2018/3901016

**Published:** 2018-03-13

**Authors:** Naoki Yamada, Ryo Ueda, Wataru Kakuda, Ryo Momosaki, Takahiro Kondo, Takuya Hada, Nobuyuki Sasaki, Takatoshi Hara, Atsushi Senoo, Masahiro Abo

**Affiliations:** ^1^Department of Rehabilitation Medicine, The Jikei University School of Medicine, 3-25-8 Nishi-Shimbashi, Minato-Ku, Tokyo 105-8461, Japan; ^2^Department of Rehabilitation Medicine, Shimizu Hospital, 129 Miyagawacho, Kurayoshi, Tottori 682-0881, Japan; ^3^Department of Radiological Sciences, Graduate School of Human Health Sciences, Tokyo Metropolitan University, 7-2-10 Higashi-Ogu, Arakawa-ku, Tokyo 116-8551, Japan

## Abstract

We aimed to investigate plastic changes in cerebral white matter structures using diffusion tensor imaging following a 15-day stroke rehabilitation program. We compared the detection of cerebral plasticity between generalized fractional anisotropy (GFA), a novel tool for investigating white matter structures, and fractional anisotropy (FA). Low-frequency repetitive transcranial magnetic stimulation (LF-rTMS) of 2400 pulses applied to the nonlesional hemisphere and 240 min intensive occupation therapy (OT) daily over 15 days. Motor function was evaluated using the Fugl-Meyer assessment (FMA) and Wolf Motor Function Test (WMFT). Patients underwent diffusion tensor magnetic resonance imaging (MRI) on admission and discharge, from which bilateral FA and GFA values in Brodmann area (BA) 4 and BA6 were calculated. Motor function improved following treatment (*p* < 0.001). Treatment increased GFA values for both the lesioned and nonlesioned BA4 (*p* < 0.05, *p* < 0.001, resp.). Changes in GFA value for BA4 of the lesioned hemisphere were significantly inversely correlated with changes in WMFT scores (*R*^2^ = 0.363, *p* < 0.05). Our findings indicate that the GFA may have a potentially more useful ability than FA to detect changes in white matter structures in areas of fiber intersection for any such future investigations.

## 1. Introduction

Previously, we presented a novel therapeutic intervention for upper limb hemiparesis due to stroke consisting of combined treatment with low-frequency repetitive transcranial magnetic stimulation (rTMS) and intensive occupational therapy (OT) [1–3]. Clinical data obtained from more than 3000 patients have indicated that this intervention safely and significantly improves motor function in the affected upper limb [1–3]. Using serial functional magnetic resonance imaging (fMRI), we subsequently observed that neural activation in the lesioned cerebral cortex contributes to functional recovery following stroke [4].

Numerous MRI studies have utilized generalized fractional anisotropy (GFA) values to obtain detailed information regarding brain structure [5]. The GFA value for a region can be obtained using diffusion tensor imaging (DTI) and is considered to reflect fiber density, axonal diameter, and myelination in the cerebral white matter. While fractional anisotropy (FA), which is calculated based on flux according to three-dimensional diffusion theory, has been popular in recent years [6–8], GFA allows researchers to obtain more detailed, multiangle diffusion information [9]. Previous studies have further demonstrated the reliability and validity of FA and GFA analyses [10].

To date, no clinical studies have utilized diffusion tensor MRI for the longitudinal investigation of changes in GFA values in patients undergoing rehabilitative intervention for the treatment of stroke-induced hemiparesis. Furthermore, the influence of repetitive transcranial magnetic stimulation (rTMS) on neural network development in the cerebral white matter has not been sufficiently studied in this patient population. Although not reported in our preliminary investigation, we observed no significant differences in FA values before and after our combined intervention (rTMS-OT therapy). While FA values can be used to represent diffusive anisotropy, these values decrease in regions of nerve fiber intersection, though no such reductions are observed for GFA values. Therefore, we performed diffusion tensor imaging to assess GFA values in patients with hemiparetic stroke both prior to and following combined rTMS-OT therapy.

## 2. Materials and Methods

### 2.1. Participants

A total of 36 patients who had experienced a stroke with upper limb hemiparesis were prospectively enrolled in the present study between March 19, 2011, and June 23, 2012. The inclusion criteria were as follows: (1) age between 18–80 years at intervention; (2) time after onset of stroke > 12 months; (3) history of a single symptomatic stroke only [no bilateral cerebrovascular lesion] and an intact cortex; (4) Brunnstrom recovery stage [11] of 3–6 for hand/fingers (at least voluntarily able to flex the fingers with/without extension); (5) no cognitive impairment, as indicated by a pretreatment Mini Mental State Examination score of at least 26; (6) no active physical or mental illness requiring medical management; (7) absence of clinical conditions for which rTMS is contraindicated according to the guidelines suggested by Wasserman [12] [e.g., cardiac pacemakers, intracranial implants, implanted medication pumps, and pregnancy]; (8) no recent history of seizures; and (9) no epileptic discharge on pretreatment electroencephalography. Patients with radiating cornification or brainstem lesions were also excluded. All patients were referred to either the Jikei University School of Medicine or Shimizu Hospital.

During hospitalization, patients underwent a 15-day combination treatment for their upper limb hemiparesis. Treatment consisted of twelve 40-minute low-frequency rTMS sessions combined with intensive OT, consisting of 120 min one-to-one training and 120 min self-training, which was provided following each rTMS. Both the treatment protocol and investigational approach for the present study were approved by the ethical committees of the Jikei University School of Medicine and Shimizu Hospital. Written informed consent was obtained from all patients prior to treatment.

The clinical characteristics of the patients are presented in Table 1. Age at admission ranged from 41 to 79 years (mean = 62.5 ± 10.2 years), and the period after stroke onset ranged from 1.6 to 21.2 years (mean = 6.4 ± 4.2 years).

### 2.2. Therapeutic Intervention for Upper Limb Hemiparesis

Combined treatment with low-frequency rTMS and intensive OT was provided daily, except for Sundays. Using a 70 mm figure-8 coil attached to a MagPro R30 stimulator (MagVenture Company, Farum, Denmark), rTMS was delivered at a frequency of 1 Hz at the site of the nonlesioned hemisphere that elicited the largest motor-evoked potentials (MEPs) in the first dorsal interosseous (FDI) muscle of the unaffected upper limb. Each 40-minute low-frequency rTMS session consisted of 2400 pulses in total. The intensity of stimulation was set at 90% of resting motor threshold for the FDI muscle. For all patients, the laterality of the nonlesioned hemisphere was confirmed based on both physical evidence (the side of hemiparesis) and pretreatment MRI data.

OT was provided immediately after the application of low-frequency rTMS and consisted of both one-to-one training and self-training. The one-to-one training program was provided by an occupational therapist and consisted of shaping techniques (e.g., reaching forward to move a cup from one place to another, writing letters and pictures using a pencil) and repetitive task practice techniques (e.g., clay squeezing and molding, pinching small coins). Based on motor function of the affected upper limb and patient lifestyle (e.g., occupation, interests, and household work), the one-to-one training program was largely tailored at admission and sometimes modified during the course by the therapist to suit the individual needs of the patient.

Self-training tasks were similar to those applied in the one-to-one training. An occupational therapist evaluated patient performance on the training tasks after each self-training session and provided relevant feedback.

### 2.3. Clinical Evaluation of Motor Function

Motor function of the affected upper limb was evaluated using the Fugl-Meyer assessment (FMA) and Wolf Motor Function Test (WMFT) [13–16], which were conducted on the day of admission and discharge (the day following the final treatment day) by an independent occupational therapist. The FMA is a performance-based tool for the quantitative assessment of various impairments following hemiparetic stroke [13, 15]. The FMA section associated with motor function of the upper limb consists of 33 items, with a maximum motor performance score of 66 points. The WMFT evaluates motor function of the upper limb according to the mean performance time on 15 timed tasks [14, 16]. Tasks requiring more than 120 seconds to complete are assigned the maximum performance time of 120 seconds.

### 2.4. MRI

All patients underwent DTI using a 1.5 T magnetic field MRI system (ECHELON Vega, Hitachi Medico, Tokyo, Japan). The following imaging parameters were used: spin-echo diffusion echo planar image; number of axes: 21; *b* value: 1000 s/mm^2^; repetition time: 5000 ms; echo time: 74.4 ms; matrix: 256 × 256 pixels; number of excitations: 1; field of view: 240 mm; slice thickness: 5 mm; slices: 30; scan time: 163.8 s; and voxel size: 0.9 × 0.9 × 5 mm. Two exercise-related white matter regions were selected as regions of interest (ROIs), including the primary motor cortex (M1, Brodmann area [BA] 4) and ventral/dorsal premotor cortices (BA6).

### 2.5. Derivation of GFA

GFA is calculated from the following expression (1):

(1) $$\begin{array}{c} GFA=\frac{std\left(ADC\right)}{rms\left(ADC\right)}=\sqrt{\frac{n_{s}\sum_{i=1}^{n_{s}}{\left(\Psi \left(u_{i}\right)-<\Psi >\right)}^{2}}{\left(n-1\right)\sum_{i=1}^{n_{s}}\Psi {\left(n_{i}\right)}^{2}}}, \end{array}$$

where *n*_*s*_ is the number of the axes to which MPG (motion probing gradient) pulses have been applied, Ψ(*u*_*i*_) is each axial ADC (apparent diffusion coefficient), and <Ψ> is the mean of each axial ADC. GFA is evaluated for each axial ADC. The direction resolution increases as the number of axes increases.

### 2.6. Analysis of GFA Value

**Figure 1:**
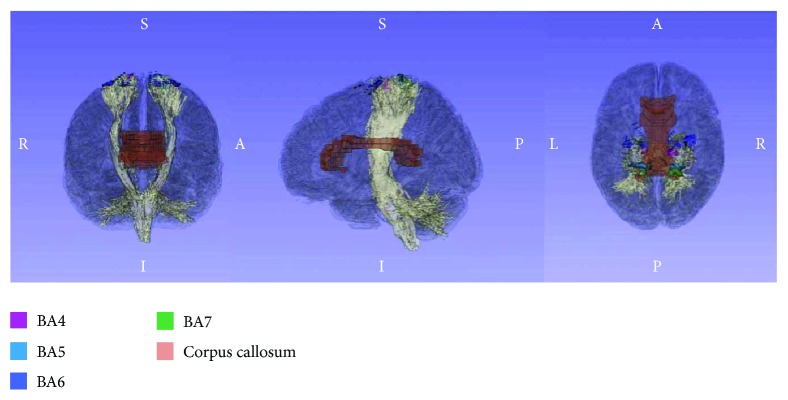
Region-of-interest (ROI) models of Brodmann areas 4 and 6. Each color represents the ROIs encompassing the white matter of Brodmann areas 4, 6, and the corpus callosum, respectively.

**Figure 2:**
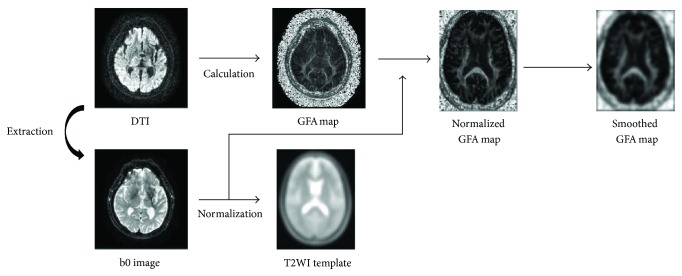
Flowchart of analysis procedures.

First, a T2-weighted image (T2WI) was extracted using IDL 6.4 (Exelis Visual Information Solutions, Boulder, CO). GFA scores were calculated by dividing the standard deviation by the root mean square (both derived from the apparent diffusion coefficient) and then visualized as a map. Images were then preprocessed using SPM8 (Wellcome Department, University College, London, UK) in MATLAB R2013a (Mathworks, Natick, MA). SPM8 is equipped with standard templates of the brain for T1WI and T2WI, but not with a standard brain map for GFA. Therefore, in the present study, we generated a standardized GFA map using the extracted T2WIs and their associated GFA maps. Smoothing was performed in SPM8 using a Gaussian filter. As noise increased following this standardization procedure, images were smoothed to improve signal-to-noise ratio. The full-width at half maximum (FMWH) was established at 6 mm. To prepare the mask images for our ROIs, we used the advanced mode in the MATLAB-compatible WFU-Pick Atlas toolbox [17, 18]. White matter and BA ROIs were chosen, and masks were made using the “INTERSECT” function in order to observe white matter within the indicated BAs. Both GFA maps after smoothing and BA mask images were coregistered to the ICBM DTI-81 of the International Consortium of Brain Mapping—a stereotaxic probabilistic white matter atlas [19]. In addition, the MRIcro program [20] was used to convert mask images to ROIs, which were prepared for white matter structures in the left and right BA4 and BA6. We loaded each BA into 3D slicer and displayed the corpus callosum and corticospinal tract as indicated in Figure 1. The average GFA within each ROI was calculated by layering the ROI over the GFA map (Figure 2). Like FA, GFA produces values from 0 to 1, with higher values denoting a greater degree of diffusion anisotropy.

### 2.7. Statistical Analysis

All statistical analyses were performed using Statistical Package for Social Sciences, version 19.0 (SPSS, Chicago, IL). Changes in FMA for upper limb motor function were examined using the Wilcoxon signed-rank test. As we obtained a skewed distribution for WMFT performance times, we used the WMFT mean performance rate data. The data were analyzed using a new calculation, which consisted of calculating the rate of task performance (60/performance time). Subjects unable to complete a task within 120 seconds were assigned a rate of 0 [21]. Furthermore, we calculated the natural logarithm of the mean performance time across the 15 timed tasks in accordance with procedures described in the EXCITE trial [22].

Changes in the WMFT mean performance rate and WMFT-log performance time (WMFT-lpt) were examined using the Wilcoxon signed-rank test. WMFT-lpt was also used to analyze the effect of treatment on FA and GFA values for each of the two ROIs. Moreover, we performed correlation analyses of changes in GFA value for each ROI in both cerebral hemispheres in relation to the extent of motor function improvement. Changes in GFA value for each ROI and all correlations were assessed using the nonparametric Spearman's rho. A *p* value of less than 0.05 was considered statistically significant.

## 3. Results

### 3.1. Changes in Motor Function

All patients completed the protocol without any adverse effects. The intervention resulted in significant improvements in motor function of the affected upper limb, as indicated FMA, WMFT. FMA scores increased from 45.4 ± 12.3 to 50.9 ± 12.0 points, while the WMFT mean performance rate increased from 24.1 ± 10.0 to 32.0 ± 13.8. In addition, WMFT-lpt decreased from 2.8 ± 1.2 to 2.4 ± 1.3 (Wilcoxon signed-rank test, all *p* values < 0.001).

### 3.2. Analysis of FA and GFA Value

As indicated in Table 2, the FA value did not significantly increased following treatment in all ROIs, whereas the GFA value significantly increased following treatment in specific ROIs (*p* = 0.037 for BA4 in the lesioned hemisphere; *p* < 0.001 for BA4 in the nonlesioned hemisphere). In addition, we observed a significant inverse correlation between changes in GFA for BA4 in the lesioned hemisphere and changes in WMFT-lpt (*R*^2^ = 0.363, *p* = 0.30).

## 4. Discussion

In the present study, we performed serial evaluation of GFA values in both the left and right hemispheres of patients treated with low-frequency rTMS and intensive OT over 15 days using a novel analytic technique based on diffusion MRI. To our knowledge, the present study is the first to investigate the influence of combined rehabilitative intervention using such methods. Our results indicate that the intervention not only improved upper limb hemiparesis but also changed the white matter structures directly under BA4 of the lesioned hemisphere, as well as under BA4 of the nonlesioned hemisphere. Furthermore, the extent of hemiparesis improvement was significantly correlated with increases in GFA value in the lesioned BA4, which suggests that recovery of motor function is at least partly due to structural changes in the cerebral white matter of the lesioned hemisphere.

FA is considered to reflect nerve fiber density, axial filament diameter, and myelination of white matter, and GFA values are similar to FA values in this respect. In addition, both FA and GFA values express the ease of movement of a water molecule in a range from 0 to 1. These values are calculated using DTI, such that a value of 0 indicates that movement of water molecules is relatively free and unaffected by a structural body (e.g., nerve fibers and medullary sheaths), whereas a higher value indicates a strong directionality imparted by the structural body on the motion of water molecules. GFA values are high for white matter due to the density of the nerve fibers, slightly lower for gray matter, and close to 0 for cerebrospinal fluid. Further research suggests that DTI is useful for the evaluation of cerebral white matter nerve fibers in patients who have experienced cerebrovascular accidents [23]. For these reasons, the present study utilized DT imaging and GFA.

In this way, GFA could reflect the state of diffusing water molecules in greater detail than FA. That is, more information about each direction of diffusion can be obtained from the GFA calculation as compared to the FA calculation, which is calculated using eigenvalues of *λ* 1, *λ* 2, and *λ* 3 [5]. In addition, in areas where two sets of nerve fibers intersect, FA is estimated to be small, resulting in an ellipsoid representation, while the GFA calculation addresses this issue. Few reports [24–28] have focused on the analysis of white matter structures using GFA, and the present study marks the first attempt to analyze the effects of intervention using TMS.

The two ROIs in the present study were chosen due to their association with functions related to voluntary movement. Namely, BA4 (the primary motor cortex) and BA6 (composed of the premotor cortex and the supplementary motor area) are associated with voluntary movement, and these areas receive inputs from BA5 and BA7, which unify information from the somatosensory optic areas. While these BAs consist of both gray and white matter, the ROIs targeted only the white matter structures. Gray matter was not included in the analysis, as the presence of many dendrites inhibits observed changes in FA or GFA. Some research has indicated that poststroke movement disorders occur due to injury to regions associated with voluntary movement [29], and that greater improvements in recovery of motor function following cerebral damage are observed in the acute phase than in the chronic poststroke phase. Such increased improvement is thought to be associated with improved blood flow to the lesion and the resulting metabolic improvements. In contrast, later motor recovery may be accomplished via plastic changes to the affected neural structures.

Changes in GFA value for BA4 (lesioned side) exhibited an inverse correlation with changes in WMFT-lpt. Using fMRI, our group previously observed that cerebral cortical activation localizes significantly to the lesioned cerebral hemisphere in patients undergoing rTMS-OT intervention [4], which suggests that motor function may improve with activation of the lesioned side.

Researchers have long acknowledged that plastic changes in the central nervous system are associated with the formation of new neural networks in stroke recovery [30, 31]. In addition, some studies have indicated that differences in the degree of recovery following cerebrovascular accident are related to the degree of injury to the associated white matter structures [7, 32, 33]. Thus, we believe that the observed association changes in white matter structures reflect the possibility of motor recovery in patients after cerebral damage.

We further attempted to examine correlational changes in the cortex and white matter and to speculate on the mechanisms underlying changes in GFA values. Diffusion of water molecules is limited by white matter structures associated with nerve fibers, primarily the medullary sheath. Thus, increases in GFA values may be due to axonal sprouting or myelination, whereas decreases may be the result of axonal degeneration. It is worth noting that, while FA values decrease at the intersection of fibers, GFA values remain unaffected. Therefore, we can infer that tissue degeneration—rather than the presence of fiber intersections—is responsible for decreases in GFA value. Furthermore, white matter degeneration is thought to be associated with functioning of nearby gray matter, as the nerve fibers convey signals from the cell's perikaryon. Therefore, we maintain that the decrease in GFA values may be directly related to white matter denaturation and plastic changes associated with regaining cortical function.

The present study has certain limitations. First, the study included only 36 patients and lacked a control group. The lack of a control group or condition, however, is mitigated by our previous study that included controls and replicated clinical findings over multiple sites [34].

Accordingly, the observed clinical improvements could be due to specific effects. In addition, the white matter changes might happen. Another factor that makes the lack of a control group a lesser limitation than otherwise is that the participants had experienced stroke > 1 year previously, and thus they were long past the time window during which spontaneous recovery takes place. Second, previous reports have used GFA on high-angular-resolution-diffusion imaging data with 200+ directions [5, 23, 35]. In this study, we calculated GFA using data obtained from 21 DTI axes due to the constraints of clinical practice, and GFA analysis was performed only for BA4 and BA6. However, as beneficial reorganization in some other cerebral areas may contribute to motor function recovery of the paretic upper limb, future studies should assess GFA changes in additional brain areas. Future studies should also examine whether certain changes in GFA and motor function are due to OT alone, rTMS alone, or the combination of interventions. Finally, long-term follow-up studies are required to determine whether changes in GFA observed following the intervention are maintained over time. Other limitations are the small proportion of women in the sample and lack of blinding. Thus, our findings should be confirmed in a randomized controlled trial with a larger number of patients in whom changes in motor function are evaluated outside of the treatment setting, that is, in everyday life.

## 5. Conclusions

The application of low-frequency rTMS combined with intensive OT resulted in increased GFA values in BA4, as well as improvements in upper limb hemiparesis, suggesting that the combined rTMS-OT intervention may induce beneficial plastic changes in cerebral white matter structures, though the lack of a control group in the present study limits this interpretation. Serial assessments of GFA values seem to provide reliable evidence of structural changes in cerebral white matter in patients undergoing rehabilitative intervention following hemiparetic stroke, although further studies are required to verify this finding.

## Figures and Tables

**Table 1:** Patient characteristics.

Characteristic	Value
*Age at intervention*, *years*	62.5 ± 10.2
*Sex*	
Female	8 (22)
Male	28 (78)
*Time after stroke*, *months*	77.3 ± 50.3
*Subtype of stroke*	
ICH	18 (50)
CI	18 (50)
*Side of hemiparesis*	
Dominant hand	20 (56)
Nondominant hand	16 (44)
*BRS for hand-fingers*	
III	4 (11)
IV	18 (50)
V	10 (28)
VI	4 (11)

Values are presented as numbers (percentage) or means ± standard deviations. ICH: intracerebral hemorrhage; CI: cerebral infarction; BRS: Brunnstrom recovery stage.

**Table 2:** Analysis of FA and GFA values.

	Preintervention FA value (mean ± SD)	Postintervention FA value (range)	*p* value	Preintervention GFA value (range)	Postintervention GFA value (range)	*p* value	Correlation between GFA change and FMA change		Correlation between GFA change and WMFT-LPT change	
							*R* value	*p* value	*R* value	*p* value
*Brodmann area 4*										
Lesioned hemisphere	0.194 ± 0.023	0.195 ± 0.025	0.762	0.181 ± 0.006	0.186 ± 0.005	0.037^∗^	−0.137	0.420	−0.363	0.030^∗^
Nonlesioned hemisphere	0.207 ± 0.018	0.211 ± 0.013	0.069	0.193 ± 0.012	0.200 ± 0.014	<0.001^∗^	−0.092	0.588	−0.059	0.734
*Brodmann area 6*										
Lesioned hemisphere	0.179 ± 0.006	0.181 ± 0.004	0.525	0.165 ± 0.0002	0.168 ± 0.001	0.093	−0.155	0.359	−0.236	0.165
Nonlesioned hemisphere	0.189 ± 0.002	0.190 ± 0.003	0.822	0.172 ± 0.007	0.175 ± 0.005	0.054	0.042	0.804	−0.269	0.112

Data are mean ± SD. ^∗^*p* < 0.05. FA: fractional anisotropy; GFA: generalized fractional anisotropy; FMA: Fugl-Meyer assessment; WMFT-LPT: Wolf Motor Function Test-log performance time.
